# *UGT1A1* genetic variants are associated with increases in bilirubin levels in rheumatoid arthritis patients treated with sarilumab

**DOI:** 10.1038/s41397-022-00269-5

**Published:** 2022-02-11

**Authors:** Nan Lin, Amy Damask, Anita Boyapati, Jennifer D. Hamilton, Sara Hamon, Nils Ternes, Michael C. Nivens, John Penn, Alexander Lopez, Jeffrey G. Reid, John Overton, Alan R. Shuldiner, Goncalo Abecasis, Aris Baras, Charles Paulding

**Affiliations:** 1 Regeneron Pharmaceuticals, Inc., Tarrytown, NY USA; 2 Translational Medicine Statistics, Sanofi R&D, Chilly-Mazarin, France; 3 Present Address: xVantage Solutions, DNAnexus, Mountain View, CA USA

**Keywords:** Genetic association study, Genotype, Sequencing, Rheumatic diseases

## Abstract

Sarilumab is a human monoclonal antibody against interleukin (IL)-6Rα that has been approved for the treatment of adult patients with moderately to severely active rheumatoid arthritis (RA) and an inadequate response or intolerance to one or more disease-modifying antirheumatic drugs (DMARDs). Mild liver function test abnormalities have been observed in patients treated with sarilumab. We describe a genome-wide association study of bilirubin elevations in RA patients treated with sarilumab. Array genotyping and exome sequencing were performed on DNA samples from 1075 patients. Variants in the *UGT1A1* gene were strongly associated with maximum bilirubin elevations in sarilumab-treated patients (rs4148325; *p* = 2.88 × 10^−41^) but were not associated with aminotransferase elevations. No other independent loci showed evidence of association with bilirubin elevations after sarilumab treatment. These findings suggest that most bilirubin increases during sarilumab treatment are related to genetic variation in *UGT1A1* rather than underlying liver injury.

## Introduction

Drug-induced liver injury (DILI) is the most frequent cause of safety-related postmarketing drug withdrawals [1, 2] and is the most common reason for acute liver failure in the United States [3, 4]. DILI shares many clinical features with other forms of liver injury, making a definitive DILI diagnosis challenging [3]. A DILI diagnosis is usually determined by ruling out other common forms of liver injury, such as gall bladder disease, malignancy, and concomitant hepatotoxic drugs [2, 3]. Alanine aminotransferase (ALT), aspartate aminotransferase (AST), and total bilirubin are routinely measured in clinical trials to detect potential signals of drug-induced liver abnormalities. Drug-induced elevations in aminotransferases >3 × the upper limit of normal (ULN) in combination with total bilirubin elevations >2 × ULN have been termed “Hy’s law”, and may be indicative of severe hepatocellular injury and DILI [2]. However, a number of drugs can cause transient aminotransferase elevations without progressing to severe liver injury (e.g., aspirin, statins, heparin) [2], and genetic factors can predispose some individuals to hyperbilirubinemia.

The uridine diphosphate (UDP) glucuronosyltransferase family 1 member A1 gene (*UGT1A1*) encodes the enzyme UDP-glucuronosyltransferase 1-1, responsible for the conjugation of bilirubin and glucuronic acid, forming a water-soluble glucuronide that is excreted into the bile [5]. Genetic variation in *UGT1A1* has been linked to Gilbert’s syndrome, a common benign condition characterized by elevations in unconjugated bilirubin and jaundice in the absence of aminotransferase elevations. The underlying genetic variation responsible for the majority of cases has been identified as a TA repeat polymorphism (UGT1A1*28 allele) located in the promoter region [6]. UGT1A1*28 carriers have seven copies of the TA repeat polymorphism [A(TA)_7_TAA] and ~70% reduced *UGT1A1* gene expression versus individuals with the more common allele featuring six copies of the TA repeat [A(TA)_6_TAA] [5]. Gilbert’s syndrome patients are typically homozygous carriers of the UGT1A1*28 allele, and have lower *UGT1A1* gene expression and diminished bilirubin metabolism. Other variants have been identified that influence *UGT1A1* function and are associated with Gilbert’s syndrome but are less common than the UGT1A1*28 allele [5, 7].

Sarilumab is a human immunoglobulin G1 monoclonal antibody that binds specifically to both soluble- and membrane-bound interleukin (IL)-6α receptors (IL-6Rα) and inhibit IL-6–mediated signaling through these receptors. Sarilumab has been approved for the treatment of adult patients with moderately to severely active rheumatoid arthritis (RA) and an inadequate response or intolerance to other disease-modifying antirheumatic drugs (DMARDs). Elevations in transaminases have been observed in sarilumab-treated patients, and it is recommended that ALT and AST levels be assessed 4 to 8 weeks after the initiation of therapy and every 3 months thereafter [8–10]. During the clinical development of sarilumab, no cases of Hy’s law were observed with treatment [10].

Two studies in patients treated with tocilizumab, an IL-6Rα inhibitor, identified a strong association between the UGT1A1*28 allele and increases in unconjugated bilirubin levels [11, 12]. We retrospectively tested if *UGT1A1* variants were associated with bilirubin elevations in sarilumab-treated patients, and performed a genome-wide association study (GWAS) to explore if any genetic variants outside of the *UGT1A1* locus were associated with bilirubin elevations. Understanding the role common *UGT1A1* genetic variation may play in serum bilirubin elevations in sarilumab-treated patients may allow physicians to more clearly differentiate true Hy’s law cases from false positives.

## Subjects and methods

### Study population

The pharmacogenomic study population included patients from three clinical trials: MOBILITY (NCT01061736) [13], TARGET (NCT01709578) [14], and ASCERTAIN (NCT01768572) [15]. These studies were randomized, double-blind, parallel-group trials investigating the efficacy and/or safety of sarilumab in adults with moderately to severely active RA. Inclusion criteria for disease history and severity were similar across studies. MOBILITY used the American College of Rheumatology (ACR) 1987 Rheumatoid Arthritis Classification Criteria to diagnose RA, while TARGET and ASCERTAIN used ACR/European League against Rheumatism 2010 Rheumatoid Arthritis Classification Criteria.

All study protocols were approved by the appropriate ethics committees/institutional review boards. A separate written informed consent was provided by patients who participated in the optional pharmacogenomics substudy. The trials were conducted in compliance with institutional review board regulations, the International Conference on Harmonization Guidelines for Good Clinical Practice, and the Declaration of Helsinki.

### Genotype and whole-exome sequencing data

Patient DNA samples underwent microarray genotyping and whole-exome sequencing. Two microarray platforms were used in these studies. The Illumina Infinium Human OmniExpress Exome Bead Chip, v1.2 (Illumina Inc., San Diego, CA, USA) generated microarray genotypes for samples from MOBILITY. The Illumina Global Screening Array, v1.0 genotyped samples from TARGET and ASCERTAIN. Additional genetic data were generated via whole-exome sequencing. DNA samples from MOBILITY underwent exome capture using the NimbleGen VCRome, while exome capture for DNA samples from TARGET and ASCERTAIN was conducted using the IDT xGen v1.0 kit. For all studies, sequencing was performed using Illumina HiSeq 2500 instruments. All whole-exome sequencing and microarray genotyping were conducted by the Regeneron Genetics Center.

Identical quality control (QC) metrics were applied to variants genotyped on the Illumina Infinium Human OmniExpress Exome array and the Illumina Global Screening Array. The call rates (% of nonmissing genotypes) by variant and by sample were calculated. Genetic variants with a call rate <90% were excluded from further statistical analyses. Individual samples with a call rate <90%, or discordance between genetically determined sex and sex in the clinical database, were removed from further analyses. Additionally, relatedness between all study participants was evaluated. Paired samples with estimated identity-by-descent ≥0.1875 were flagged as potential duplicates/familial relationships; the sample with the lower call rate was removed from the analysis.

Hardy–Weinberg equilibrium (HWE) tests were calculated in the major genetically determined ancestry subgroups. Variants with significant deviations from HWE (*p* < 1 × 10^−6^) were removed from the analysis. Genotype imputation with array data was used to infer additional genotypes using the program Minimac4 through the Michigan Imputation Server [16]. Reference populations for imputation were obtained from the 1000 Genomes phase 3 version 5 data [17]. Additional QC measures were applied to imputed genotypes, including a minor allele frequency ≥1%, missingness < 0.1, and estimated imputation *r*^2^ (rsq-hat) ≥ 0.3.

Population structure was assessed using principal components analysis (PCA) with PLINK software (versions 1.9 and 2) [18]. Each patient was assigned to one of five major ancestral classes (African, Admixed American, East Asian, European, or South Asian) based on the similarity between each patient’s genotypes and genetic data from the International HapMap project. Principal components were calculated for each of the HapMap groups of known ancestry. A kernel density estimate was trained for each ancestral class, and the likelihood of each patient belonging to one of the five major classes was calculated to assign ancestral class.

### Statistical analysis

Due to the small patient numbers in each study, data were pooled, and all analyses included a covariate for study group. Unless otherwise specified, all postbaseline analyses were stratified by treatment group (i.e., placebo + DMARD or sarilumab + DMARD).

The primary purpose of this analysis was to describe a genome-wide association of bilirubin elevations in RA patients treated with sarilumab. The analysis endpoints included total bilirubin (mg/dl), unconjugated bilirubin (mg/dl), conjugated bilirubin (mg/dl), ALT (ukat/l), and AST (ukat/l) and alkaline phosphatase (ALP). All endpoints were measured longitudinally over the 24-week study periods, at 2-week intervals during the first 12 weeks and at 4-week intervals thereafter. All patients had measurements at the baseline study visits. Following the clinical study statistical analysis methods, the last observation carried forward approach was used to impute postbaseline missing values. During the clinical trials, drug treatment was discontinued in cases of ALT >5 × ULN or ALT >3 × ULN and concomitant total bilirubin >2 × ULN (unless the patient had documented Gilbert’s syndrome), however, no cases of Hy’s law were observed [10]. In cases of ALT ≥3 × but ≤5 × ULN and bilirubin ≤2 × ULN, treatment was withheld, and ALT/AST and bilirubin levels were measured within 48 h of the original laboratory results.

In the GWAS analysis, a linear regression model was used to evaluate the association between each endpoint and variant genotypes. The model included study, gender, age, baseline measurement (in all postbaseline analyses), and the first eight PCA scores as covariates. Due to the skewed distribution observed for maximum bilirubin, this endpoint was transformed with a rank inverse normal transformation prior to genetic analysis.

To assess the association between each endpoint and candidate variants in the *UGT1A1* gene region, we applied functional canonical correlation analysis (FCCA). The mathematical and computational details of FCCA have been described previously [19]. In this analysis, the variant is treated as the X matrix, while the longitudinal endpoint data are treated as the Y matrix. Time interval information is incorporated in the model to globally test (across all time points) whether variant genotypes are associated with the endpoint. For all the FCCA tests, baseline measurement, study, gender, age, and first eight PCA scores were included as covariates to adjust for potential confounding factors. To further investigate the total bilirubin elevation in sarilumab-treated patients, total bilirubin level was dichotomized into two categories by the ULN level (≤1.5 × ULN versus >1.5 × ULN; 1.5 × ULN of total bilirubin corresponds to 1.84 mg/dl). Firth logistic regression, assuming a recessive genetic model, was used to test the association between the candidate variants in *UGT1A1* and ULN-dichotomized total bilirubin level in sarilumab-treated patients; covariate adjustment followed the approach outlined above. Fisher’s exact tests were applied to confirm results of any Firth regression models.

### Software

All summaries and statistical analyses were generated using R (version 3.4.1 or higher) [20], Python (2.7.16) [21], and PLINK (versions 1.9 and 2) [22]. QCtools v2 and VCFtools were used to reformat genetic data. Summaries of genetic data and additional QC checks of genotype and sequence data were conducted using PLINK. Genotype data prephasing and imputation were computed at the Michigan Imputation Server [16] with 1000 Genomes Phase 3 version 5 as the reference panel [17].

## Results

A total of 1075 patients (642, 368, and 65 from MOBILITY, TARGET, and ASCERTAIN, respectively) provided DNA and consented to participate in the pharmacogenomics analysis. Patients’ ancestry was determined using principal components as shown in Supplementary Fig. 1. In total, 55% of patients were assigned European ancestry, 36% were assigned Admixed American ancestry (due to a large number of clinical sites in South and Central America), and 2% were assigned to other ancestries (Table 1). At baseline, patients from MOBILITY and TARGET had similar levels of bilirubin and transaminases. Patients from ASCERTAIN had slightly higher levels of bilirubin at baseline, but they represented only 6% of the patients in the pharmacogenomic analysis. Due to the treatment allocation design of the clinical trials, 755 (70%) patients were treated with sarilumab + DMARD, while 320 (30%) patients were treated with placebo + DMARD.

**Table 1:** Patient demographic and baseline characteristics in pharmacogenomics analysis.

Characteristic	MOBILITY (*n* = 642)	TARGET (*n* = 368)	ASCERTAIN (*n* = 65)	Total (*n* = 1075)
Genetically determined ancestry, *n* (%)				
European	362 (56)	189 (51)	46 (71)	597 (55)
Admixed American	206 (32)	158 (43)	18 (27)	382 (36)
African	23 (4)	15 (4)	1 (2)	39 (4)
Asian	32 (5)	6 (2)	0 (0)	38 (4)
Other	19 (3)	0 (0)	0 (0)	19 (2)
Treatment status, *n* (%)				
Sarilumab + DMARD	440 (69)	250 (68)	65 (100)	755 (70)
Placebo + DMARD	202 (31)	118 (32)	0 (0)	320 (30)
Age, mean years ± SD	51.13 ± 12.32	53.07 ± 12.4	52.8 ± 12.41	52.8 ± 12.41
Gender, *n* (%)				
Male	134 (21)	62 (17)	14 (22)	210 (20)
Female	508 (79)	306 (83)	51 (78)	865 (80)
Baseline biomarkers, mean ± SD				
Total bilirubin, mg/dl	0.39 ± 0.18	0.38 ± 0.17	0.45 ± 0.20	0.39 ± 0.18
Conjugated bilirubin, mg/dl	0.10 ± 0.05	0.10 ± 0.04	0.12 ± 0.06	0.10 ± 0.05
Unconjugated bilirubin, mg/dl	0.26 ± 0.16	0.25 ± 0.15	0.31 ± 0.17	0.26 ± 0.16
Alanine transaminase, ukat/l	0.36 ± 0.24	0.36 ± 0.22	0.36 ± 0.19	0.36 ± 0.22
Aspartate transaminase, ukat/l	0.36 ± 0.17	0.35 ± 0.16	0.39 ± 0.13	0.36 ± 0.16

*DMARD* disease-modifying antirheumatic drug, *n* number of participants, *SD* standard deviation.

### Associations between bilirubin levels and *UGT1A1* variants

**Fig. 1:**
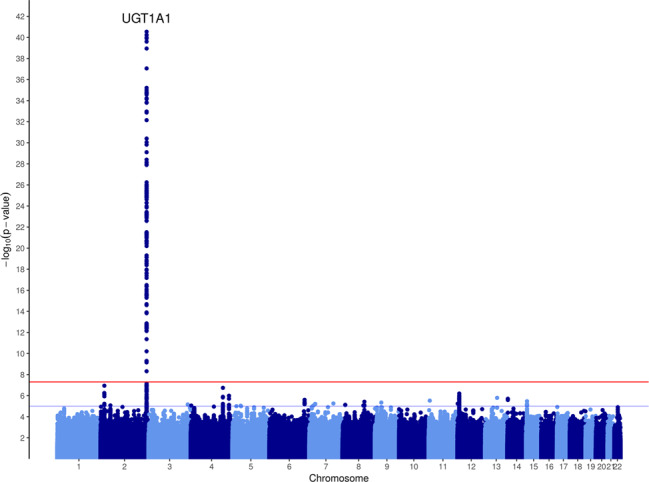
Manhattan plot of the genome-wide association analysis of maximum total bilirubin in sarilumab + DMARD-treated patients (*N* = 755) during the treatment periods. The most significant variant was in the *UGT1A1* gene (rs4148325; *p* = 2.88 × 10^−41^).

**Fig. 2:**
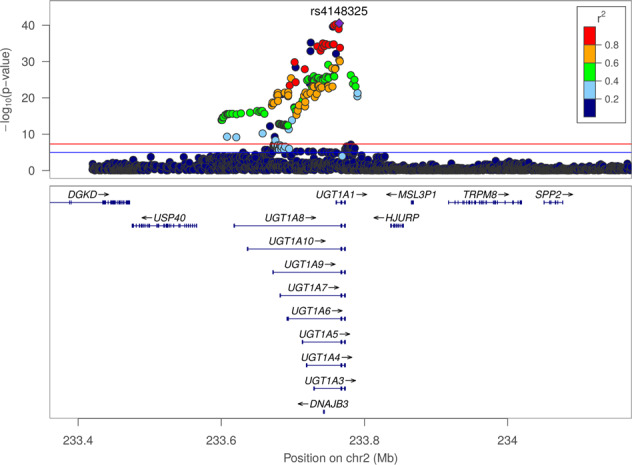
LocusZoom plot of the top loci identified in genome-wide association analysis according to maximum total bilirubin of sarilumab + DMARD-treated patients (*N* = 755) during the treatment periods. The upper panel shows the top variant rs4148325 (indicated with a purple diamond) at position chr2:233764663 with *p* = 2.88 × 10^−41^. The other variants are represented by colored circles based on their level of correlation with rs4148325: red indicates strong correlation, and blue indicates weak or no correlation. The lower panel shows that the *UGT1A* gene locus is comprised of several family members that share exons 2–5 but have a unique exon 1 [5].

The GWAS of maximum bilirubin levels during the study period in patients treated with sarilumab + DMARD identified a strong association on chromosome 2 (Fig. 1) localized to the *UGT1A1* gene (Fig. 2) (significant variants, Supplementary Table 1) (QQ plot, Supplementary Fig. 2). All postbaseline analyses were adjusted for baseline bilirubin. The top variant, rs4148325 (*β* = 0.28 mg/dl per T allele increase; *p* = 2.88 × 10^−41^), had mean (SE) bilirubin levels of 0.57 (0.01) mg/dl, 0.66 (0.01) mg/dl, and 1.19 (0.05) mg/dl in the CC, CT, and TT genotypes, respectively. Variant rs4148325 is in the intronic region of the *UGT1A1* gene as well as several related family members in the *UGT1A* locus (Fig. 2). This variant is ~4.4 kb from the well-characterized UGT1A1*28 TA repeat (rs3064744). While the UGT1A1*28 variant was not directly genotyped or imputed in our dataset, previous studies have reported rs4148325 to be in strong linkage disequilibrium with UGT1A1*28 (*r*^2^ = 0.88 and *D*′ = 0.98) [11]. Additionally, we confirmed that rs4148325 was the most significant variant in change from baseline analyses at Weeks 2, 12, and 24, with *p* values ranging from 4.46 × 10^−10^ to 1.16 × 10^−17^. Across all analyses, there were no variants with genome-wide significant *p* values (<5 × 10^−8^) outside of the *UGT1A1* region.

**Fig. 3:**
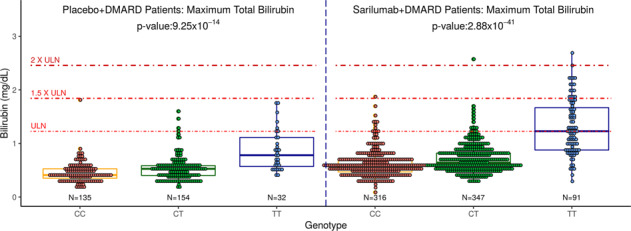
Maximum total bilirubin level (mg/dl) by UGT1A1 (rs4148325) genotype and treatment (placebo + DMARD or sarilumab + DMARD). Boxplots of the maximum bilirubin level by genotype are shown.

Maximum bilirubin level by rs4148325 genotype was assessed in placebo + DMARD-treated patients. While significant, the effect size and *p* value were of lesser magnitude (*β* = 0.16 mg/dl per allele; *p* = 9.25 × 10^−14^) than the effects observed in sarilumab + DMARD-treated patients. The genotypic means (SE) were comparatively lower: 0.46 (0.02) mg/dl; 0.52 (0.02) mg/dl; and 0.87 (0.06) mg/dl in the CC, CT, and TT genotypes, respectively. The persistent association of bilirubin and rs4148325 genotype in the absence of sarilumab treatment may be due to the underlying DMARD use. A comparison of maximum bilirubin levels during the clinical trial period, stratified by treatment arm, is shown in Fig. 3.

**Fig. 4:**
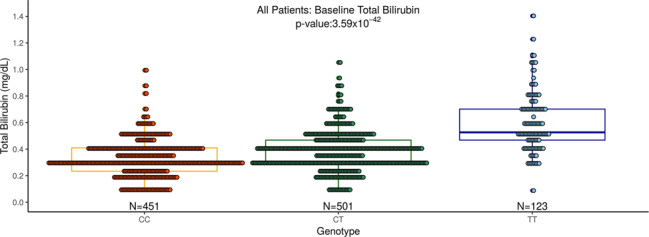
Baseline total bilirubin levels (mg/dl) by UGT1A1 (rs4148325) genotype for all patients (placebo + DMARD and sarilumab + DMARD arms are combined). Boxplots of the baseline bilirubin level by genotype are shown. Treatment arms were combined to assess bilirubin levels pre-treatment.

To further evaluate the association between rs4148325 and bilirubin levels, an analysis at baseline in all patients was conducted. This also demonstrated a strong association (Fig. 4; *p* = 3.59 × 10^−42^, *β* = 0.11 mg/dl per allele). Mean (SE) baseline bilirubin was 0.33 (0.007), 0.38 (0.007), and 0.60 (0.02) mg/dl in the CC, CT, and TT genotypes, respectively.

To assess the potential clinical impact of the variability in bilirubin level by rs4148325 genotype, analysis of bilirubin elevations with respect to ULN were conducted. By study entry criteria, no patients had bilirubin elevations in excess of 1.5 × ULN at baseline. Additionally, no elevations >1.5 × ULN in bilirubin were observed in placebo+DMARD-treated patients, while 18 sarilumab-treated patients had bilirubin elevations >1.5 × ULN. In the binary analysis of maximum total bilirubin among sarilumab-treated patients, the TT genotype was significantly associated with bilirubin elevations (Table 2; odds ratio 47.79; *p* = 5.67 × 10^−10^). This finding was confirmed with a Fisher’s exact test (*p* = 1.14 × 10^−47^). In total, 18 of 755 (2.4%) of sarilumab-treated patients had bilirubin elevations >1.5 × ULN, and of those, 16 of 18 (89%) were homozygous for the T allele, while only two patients (11%) were in either the CC or CT genotype group. Only 2 of 664 (0.3%) CC or CT patients had bilirubin elevations >1.5 × ULN compared to 16 of 91 (17.6%) of TT patients with bilirubin elevations >1.5 × ULN (Table 2).

**Table 2:** Maximum total bilirubin in sarilumab-treated by *UGT1A1* (rs4148325) genotype.

Genotype	≤1.5 × ULN, *n* (%)	>1.5 × ULN, *n* (%)	Total, *n* (%)
CC/CT	662 (90)	2 (11)	664 (88)
TT	75 (10)	16 (89)	91 (12)
Total	737	18	755

OR = 47.79 and *p* = 5.67 × 10^−10^.

*n* number of participants, *OR* odds ratio, *ULN* upper limit of normal.

**Fig. 5:**
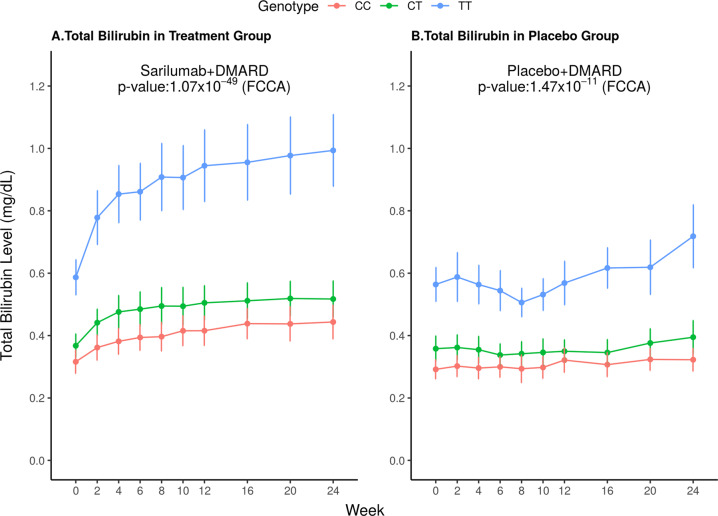
Change in total bilirubin level (mg/dl) over time by UGT1A1 (rs4148325) genotype in A Sarilumab + DMARD-treated patients and B Placebo + DMARD-treated patients. Functional canonical correlation analysis (FCCA) was applied to test the association between the change in total bilirubin level over time and rs4148325 genotype (Interaction: rs4148325 genotype × treatment, *p* = 0.03).

Longitudinal analysis of bilirubin levels measured at several time points during the 24-week treatment periods demonstrated differences between genotype groups over the course of the study (Fig. 5). Patients treated with sarilumab+DMARD in the TT genotype group showed the greatest increase in bilirubin levels over time (*β* = 0.09 mg/dl; *p* = 1.07 × 10^−49^; treatment interaction *p* = 0.03). This analysis was extended to measurements in both unconjugated bilirubin (Supplementary Fig. 3) and conjugated bilirubin (Supplementary Fig. 4). The increases in unconjugated bilirubin were significant (*β* = 0.08 mg/dl; *p* = 6.76 × 10^−45^) and greater than increases in conjugated bilirubin, suggesting that most of the increase in total bilirubin is unconjugated. However, conjugated bilirubin levels were also increased in the TT genotype group (*β* = 0.01 mg/dl; *p* = 4.39 × 10^−30^).

We also observed 29 carriers of the UGT1A1*6 allele (G71R). This variant has been shown in previous studies to have decreased *UGT1A1* function [7]. This variant was nominally associated with total bilirubin at baseline (*p* = 3.5 × 10^−3^) but was not significantly associated with bilirubin elevations in sarilumab-treated patients (*p* = 0.33) (Supplementary Table 2). Three other protein coding variants were observed in our dataset (E180E, P364L, P476P), but were too rare for inferential analysis (Supplementary Table 2).

### Comparison of ALT, AST and ALP levels for the rs4148325 variant

Additional analyses were performed for the rs4148325 variant comparing ALT, AST and ALP levels across each genotype group for evidence of underlying liver toxicity. Analysis of ALT elevations during the 24-week treatment periods for sarilumab-treated patients and placebo patients by rs4148325 genotype showed no evidence of association (Supplementary Fig. 5). Similarly, no association was observed for AST levels during the treatment period (Supplementary Fig. 6). None of the 16 sarilumab-treated patients with >1.5 × ULN bilirubin levels in the TT genotype group had elevations of ALT or AST >3 × ULN. The absence of ALT or AST elevations >3 × ULN in patients with the TT genotype suggests there was no underlying liver toxicity during treatment. Similarly, no significant association was observed between ALP elevations and rs4148325 in sarilumab-treated patients with a *p* value of 0.89.

## Discussion

We identified a strong statistical association between bilirubin elevations and *UGT1A1* gene variants in sarilumab-treated RA patients. Sarilumab-treated patients homozygous for the rs4148325 T allele had significant unconjugated and conjugated bilirubin elevations; specifically sarilumab-treated patients homozygous for the T allele had maximum total bilirubin levels of 1.30 (0.05) mg/dl compared with 0.87 (0.03) mg/dl for patients receiving placebo. These values are in line with benign elevations in total bilirubin seen in Gilbert’s syndrome. Due to the strong linkage disequilibrium between the rs4148325 variant and the UGT1A1*28 allele (*r*^2^ = 0.88 and *D*′ = 0.98) [11], the majority of rs4148325 TT homozygotes likely have Gilbert’s syndrome, with lower gene expression and diminished bilirubin metabolism. The highest elevations were observed in unconjugated bilirubin, consistent with decreased *UGT1A1* activity in UGT1A1*28 homozygous patients. UDP-glucuronosyltransferase 1-1, the enzyme encoded by the *UGT1A1* gene, is the only known enzyme responsible for bilirubin metabolism [23]. Consistent with this, our GWAS failed to identify any other loci outside of the *UGT1A1* gene region associated with sarilumab-related bilirubin elevations. The variation in bilirubin levels and its association with SNPs belonging to *UGT1A1* (rs4148325) and *SCLO1B1* (rs4149056) loci is reported in previous studies [24]. The top variant rs4149056 in *SLC01B1* on chromosome 12 had a *p* value of 0.1 in this study. Increase in conjugated bilirubin could be due to treatment with DMARD (methotrexate). Previous studies of methotrexate use in pediatric leukemia have also shown increase in total and direct bilirubin [25].

Our findings are consistent with previous observations in patients treated with tocilizumab, another IL-6Rα inhibitor, and suggest that IL-6 pathway inhibition leads to decreased UDP-glucuronosyltransferase 1-1 activity in Gilbert’s syndrome patients [11, 12]. The definitive mechanism by which blocking IL-6 signaling leads to the inhibition or saturation of UDP-glucuronosyltransferase 1-1 activity is unclear, but the parallel findings in studies of two IL-6Rα inhibitors suggest a class effect. As neither sarilumab nor tocilizumab are directly glucuronidated by UDP-glucuronosyltransferase 1-1, inhibition of the IL-6 pathway may indirectly affect levels of the *UGT1A1* transcript. Previous studies have shown that IL-6 can affect *UGT1A1* mRNA levels in rat hepatocytes [26]. An alternative hypothesis is that IL-6R inhibition corrects hepcidin induced hypoferremia [27] and this increased hemoglobin turnover in patients with *UGT1A1* mutations causes a corresponding increase in unconjugated bilirubin that saturates renal clearance. The exact mechanism for the increases in bilirubin for Gilbert’s syndrome patients after sarilumab treatment remains unclear and will require additional studies.

During the clinical trials, transaminase elevations occurred more frequently in sarilumab + DMARD-treated patients versus placebo+DMARD-treated patients [13]. However, the mean level of these elevations remained within the normal range, and most elevations were mild and self-limiting [8]. ALT or AST elevations >3 × ULN were not observed in sarilumab-treated patients homozygous for the rs4148325 T allele with bilirubin elevations >1.5 × ULN (*n* = 16). Consequently, no cases of Hy’s law were observed in the rs4148325 TT homozygote group, nor were any observed in the overall clinical trials for sarilumab-treated patients [10].

UGT1A1 is involved in the direct glucuronidation of a number of drugs or their metabolites, including simvastatin, ibuprofen, and irinotecan [5]. For irinotecan and atazanavir, variants in *UGT1A1* have been associated with treatment-related adverse events. Patients treated with irinotecan who are homozygous for the UGT1A1*28 allele are at greater risk of developing severe neutropenia after treatment [28]. However, these adverse events are due to decreased glucuronidation and clearance of irinotecan’s active metabolite SN-38. The neutropenia observed in irinotecan-treated patients is directly related to SN-38 activity and is not related to bilirubin metabolism nor does it share any similarities with the mild bilirubin elevations observed in sarilumab-treated patients. Atazanavir treatment can cause severe hyperbilirubinemia in some patients, with 40% reporting bilirubin levels >2.5 × ULN (Grade 3), and ≈4 to 8% reporting bilirubin levels ≥5 × ULN (Grade 4) [7]. Hyperbilirubinemia seen in atazanavir-treated patients is more common and reaches considerably higher levels than the mild elevations observed in sarilumab-treated patients. Only 2.4% of sarilumab-treated patients had bilirubin elevations >1.5 × ULN and none had Grade 3 or 4 hyperbilirubinemia.

Our findings suggest that most sarilumab-related increases in bilirubin levels are caused by common genetic variation in *UGT1A1* and not underlying severe liver injury. Gilbert’s syndrome patients, who have preexisting lower bilirubin metabolism, are susceptible to mild elevations in serum bilirubin after sarilumab treatment. However, the incorporation of genotyping data into patient safety assessments, particularly related to bilirubin elevations, may allow for more accurate diagnosis of liver safety events for patients receiving IL-6Rα inhibitors.

## Supplementary information

Supplementary tables

Supplementary figure 1

Supplementary figure 2

Supplementary figure 3

Supplementary figure 4

Supplementary figure 5

Supplementary figure 6
