# Supplementary material for: UGT1A1 genetic variants are associated with increases in bilirubin levels in rheumatoid arthritis patients treated with sarilumab
Source: Pharmacogenomics J. 2022 Feb 11;22(3):160–5. doi: 10.1038/s41397-022-00269-5 (PMC9151390; doi:10.1038/s41397-022-00269-5)
Supplement: Supplementary file 1 — Supplementary tables [file 41397_2022_269_MOESM1_ESM.docx]

**Table S1:** Top 50 significant variants of *UGT1A1* associated with bilirubin phenotype identified using GWAS

| **Chr** | **Position** | **ID** | **REF** | **ALT** | **AAF** | **MAC** | **Associated p-value** | | |
| --- | --- | --- | --- | --- | --- | --- | --- | --- | --- |
|  |  |  |  |  |  |  | **Total bilirubin (Maximum)** | **Unconjugated bilirubin (Maximum)** | **Conjugated bilirubin (Maximum)** |
| 2 | 233764663 | rs4148325 | C | T | 0.3508 | 529 | 2.9 × 10−41 | 3.7 × 10−41 | 9.1 × 10−24 |
| 2 | 233759924 | rs887829 | C | T | 0.3497 | 528 | 5.9 × 10−41 | 7.5 × 10−41 | 9.7 × 10−24 |
| 2 | 233763993 | rs6742078 | G | T | 0.3490 | 527 | 1.0 × 10−40 | 1.3 × 10−40 | 1.1 × 10−23 |
| 2 | 233757337 | rs111741722 | A | G | 0.3510 | 530 | 1.2 × 10−40 | 1.1 × 10−40 | 1.3 × 10−23 |
| 2 | 233755940 | rs35754645 | ATC | A | 0.3509 | 527 | 2.5 × 10−40 | 2.0 × 10−40 | 1.8 × 10−23 |
| 2 | 233764076 | rs4148324 | T | G | 0.3508 | 529 | 1.1 × 10−39 | 7.0 × 10−40 | 8.0 × 10−23 |
| 2 | 233764593 | 2:233764593:A:G | G | A | 0.3236 | 488 | 8.8 × 10−38 | 5.0 × 10−37 | 5.9 × 10−23 |
| 2 | 233725006 | rs112132688 | G | A | 0.3258 | 464 | 6.1 × 10−36 | 3.1 × 10−35 | 2.7 × 10−22 |
| 2 | 233741916 | rs34352510 | T | C | 0.3229 | 485 | 9.9 × 10−36 | 2.9 × 10−35 | 7.2 × 10−22 |
| 2 | 233745803 | rs11695484 | A | G | 0.3207 | 483 | 1.5 × 10−35 | 4.4 × 10−35 | 4.5 × 10−22 |
| 2 | 233757136 | rs10929302 | G | A | 0.3199 | 478 | 1.8 × 10−35 | 6.5 × 10−35 | 5.5 × 10−22 |
| 2 | 233755708 | rs6747843 | G | A | 0.3200 | 480 | 2.0 × 10−35 | 7.1 × 10−35 | 6.6 × 10−22 |
| 2 | 233756119 | rs6714634 | T | C | 0.3200 | 480 | 2.0 × 10−35 | 7.1 × 10−35 | 6.6 × 10−22 |
| 2 | 233750415 | rs2885296 | A | C | 0.3191 | 478 | 2.7 × 10−35 | 8.3 × 10−35 | 7.9 × 10−22 |
| 2 | 233744071 | rs17864701 | C | T | 0.3196 | 480 | 5.9 × 10−35 | 1.7 × 10−34 | 1.1 × 10−21 |
| 2 | 233734192 | rs7567468 | C | T | 0.3182 | 476 | 7.0 × 10−35 | 2.1 × 10−34 | 1.3 × 10−21 |
| 2 | 233740656 | rs17862875 | G | A | 0.3198 | 479 | 1.5 × 10−34 | 3.9 × 10−34 | 2.1 × 10−21 |
| 2 | 233765830 | rs929596 | A | G | 0.3123 | 471 | 1.6 × 10−34 | 1.1 × 10−33 | 5.0 × 10−22 |
| 2 | 233738671 | rs6722076 | G | A | 0.3152 | 469 | 1.1 × 10−33 | 2.1 × 10−33 | 8.8 × 10−21 |
| 2 | 233724596 | rs202203863 | C | G | 0.3418 | 486 | 1.4 × 10−33 | 6.1 × 10−33 | 5.6 × 10−21 |
| 2 | 233760233 | rs34983651 | C | CAT | 0.3281 | 462 | 7.1 × 10−33 | 3.4 × 10−33 | 8.6 × 10−18 |
| 2 | 233764816 | rs4148326 | T | C | 0.4814 | 724 | 4.0 × 10−31 | 9.0 × 10−31 | 3.7 × 10−19 |
| 2 | 233765606 | rs4663971 | C | G | 0.4838 | 719 | 8.9 × 10−31 | 1.7 × 10−30 | 1.6 × 10−19 |
| 2 | 233702448 | rs17863787 | T | G | 0.3113 | 470 | 1.5 × 10−30 | 1.5 × 10−31 | 7.0 × 10−18 |
| 2 | 233749337 | rs10179091 | T | C | 0.4793 | 717 | 7.9 × 10−30 | 1.2 × 10−29 | 2.6 × 10−18 |
| 2 | 233703807 | rs34781889 | CTCTG | C | 0.3080 | 462 | 4.0 × 10−29 | 4.2 × 10−30 | 4.4 × 10−17 |
| 2 | 233755003 | rs10929301 | C | G | 0.4914 | 739 | 7.0 × 10−29 | 1.6 × 10−28 | 7.3 × 10−18 |
| 2 | 233759599 | rs759174 | A | C | 0.4900 | 738 | 7.1 × 10−29 | 1.7 × 10−28 | 8.4 × 10−18 |
| 2 | 233758936 | rs3755319 | A | C | 0.4907 | 740 | 1.1 × 10−28 | 2.4 × 10−28 | 7.3 × 10−18 |
| 2 | 233716651 | rs6744284 | C | T | 0.3351 | 504 | 1.3 × 10−28 | 1.8 × 10−28 | 8.3 × 10−18 |
| 2 | 233780897 | rs6722064 | C | G | 0.2952 | 428 | 5.7 × 10−27 | 7.9 × 10−28 | 1.1 × 10−17 |
| 2 | 233757013 | rs4124874 | T | G | 0.5020 | 758 | 9.3 × 10−27 | 1.6 × 10−26 | 4.4 × 10−17 |
| 2 | 233729157 | rs3821242 | T | C | 0.4861 | 734 | 1.1 × 10−26 | 1.2 × 10−26 | 2.4 × 10−17 |
| 2 | 233757815 | rs4399719 | T | G | 0.5000 | 753 | 1.4 × 10−26 | 2.2 × 10−26 | 6.1 × 10−17 |
| 2 | 233746657 | rs4663333 | G | T | 0.5020 | 757 | 1.5 × 10−26 | 2.1 × 10−26 | 7.4 × 10−17 |
| 2 | 233749604 | rs7556676 | A | G | 0.4548 | 684 | 2.0 × 10−26 | 1.0 × 10−25 | 3.3 × 10−17 |
| 2 | 233746667 | 2:233746667:A:C | C | A | 0.4556 | 687 | 2.2 × 10−26 | 1.0 × 10−25 | 3.6 × 10−17 |
| 2 | 233735775 | rs2363116 | C | G | 0.4947 | 743 | 3.0 × 10−26 | 4.4 × 10−26 | 7.5 × 10−17 |
| 2 | 233733262 | rs11685892 | T | A | 0.4913 | 738 | 3.3 × 10−26 | 6.2 × 10−26 | 1.2 × 10−16 |
| 2 | 233749977 | rs2221198 | G | A | 0.4554 | 684 | 3.7 × 10−26 | 1.7 × 10−25 | 4.8 × 10−17 |
| 2 | 233735103 | rs4467260 | C | T | 0.4987 | 749 | 3.9 × 10−26 | 5.0 × 10−26 | 2.0 × 10−16 |
| 2 | 233697516 | rs6715829 | A | T | 0.3317 | 479 | 4.0 × 10−26 | 1.4 × 10−27 | 1.0 × 10−15 |
| 2 | 233728546 | rs2008595 | C | T | 0.4987 | 751 | 4.3 × 10−26 | 5.3 × 10−26 | 1.3 × 10−16 |
| 2 | 233740927 | rs10714492 | GT | G | 0.5000 | 746 | 5.1 × 10−26 | 6.4 × 10−26 | 8.6 × 10−17 |
| 2 | 233726821 | rs4294999 | A | G | 0.4954 | 747 | 5.5 × 10−26 | 7.0 × 10−26 | 8.1 × 10−17 |
| 2 | 233726270 | rs6711351 | A | G | 0.4954 | 747 | 5.5 × 10−26 | 7.0 × 10−26 | 8.1 × 10−17 |
| 2 | 233721797 | rs7597496 | A | G | 0.4993 | 752 | 6.7 × 10−26 | 8.0 × 10−26 | 2.1 × 10−16 |
| 2 | 233728923 | rs3806597 | A | G | 0.4960 | 745 | 6.9 × 10−26 | 7.6 × 10−26 | 3.6 × 10−16 |
| 2 | 233744546 | rs6741669 | A | G | 0.5020 | 752 | 7.3 × 10−26 | 1.1 × 10−25 | 1.5 × 10−16 |
| 2 | 233744538 | rs4663967 | A | C | 0.5020 | 752 | 7.3 × 10−26 | 1.1 × 10−25 | 1.5 × 10−16 |

*AAF* alternative allele frequency, *ALT* alanine aminotransferase, *Chr* chromosome, *ID* identification, *GWAS* genome-wide association study, *MAC* minor allele count, *REF* reference allele.

**Table S2:** UGT1A1 Protein Coding Variants Association Results

| ID | Annotation | Amino Acids | REF | ALT | AAF^*^ | MAC^†^ | P-value | TBL (mg/dL)^‡^ |
| --- | --- | --- | --- | --- | --- | --- | --- | --- |
| rs4148323 | missense | G71R | G | A | 0.02 | 29 | 0.33 | 0.74 |
| rs148755655 | synonymous | E180E | A | G | 6.6 × 10^-4^ | 1 | NA | 0.70 |
| rs34946978 | missense | P364L | C | T | 1.3 × 10^-3^ | 2 | NA | 0.85 |
| 2:233772385:T:C | synonymous | P476P | C | T | 6.6 × 10^-4^ | 1 | NA | 0.76 |

^*^AAF: Alternative Allele Frequency

^†^MAC: Minor Allele Count

^‡^TBL: Total Bilirubin Level in patients with selected variant

NA: Data with MAC < 10 are excluded from the analysis
