# Supplementary figures and images for: UGT1A1 genetic variants are associated with increases in bilirubin levels in rheumatoid arthritis patients treated with sarilumab
Source: Pharmacogenomics J. 2022 Feb 11;22(3):160–5. doi: 10.1038/s41397-022-00269-5 (PMC9151390; doi:10.1038/s41397-022-00269-5)

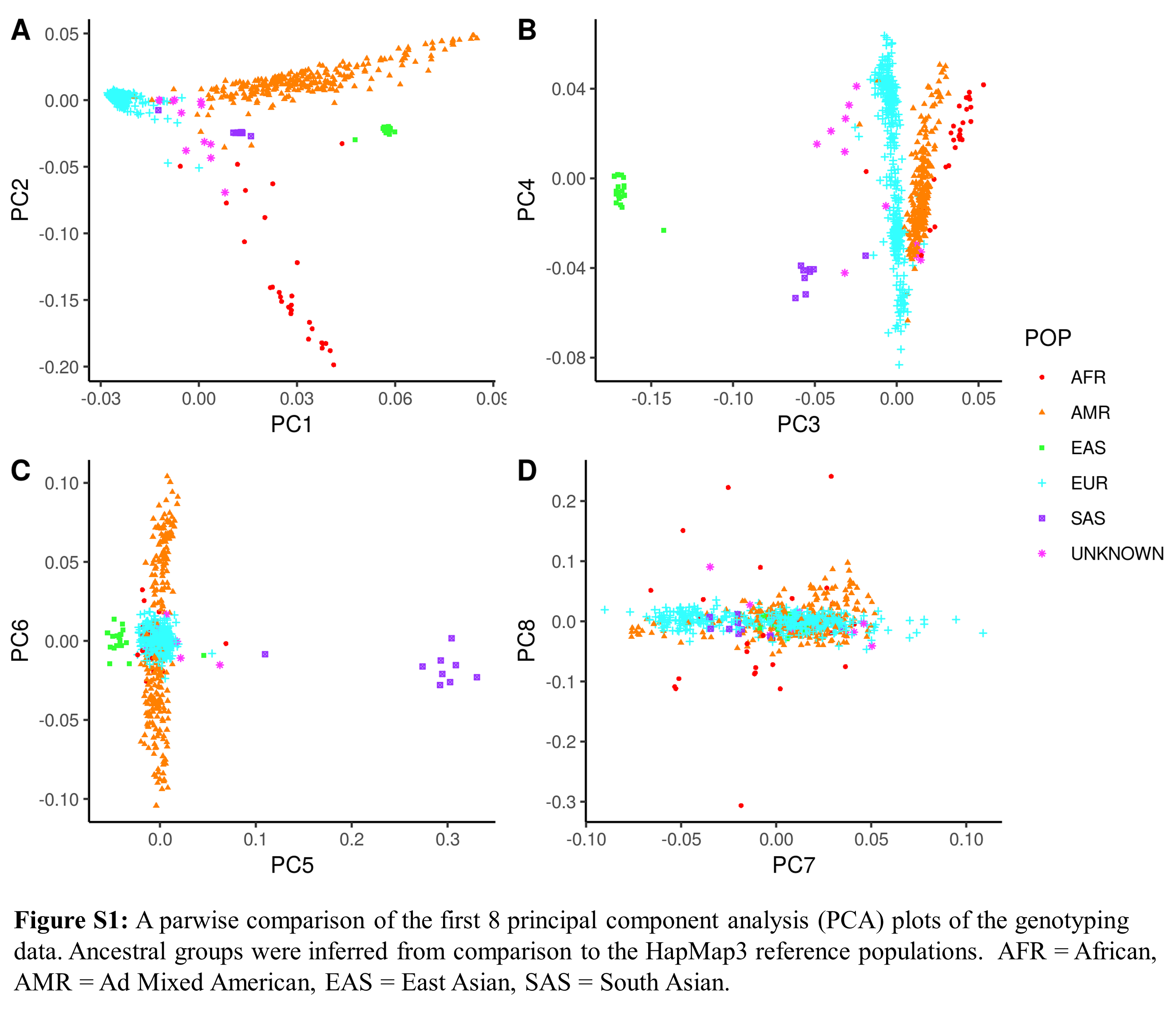

Supplement: Supplementary file 2 — Supplementary figure 1 [file 41397_2022_269_MOESM2_ESM.tif]

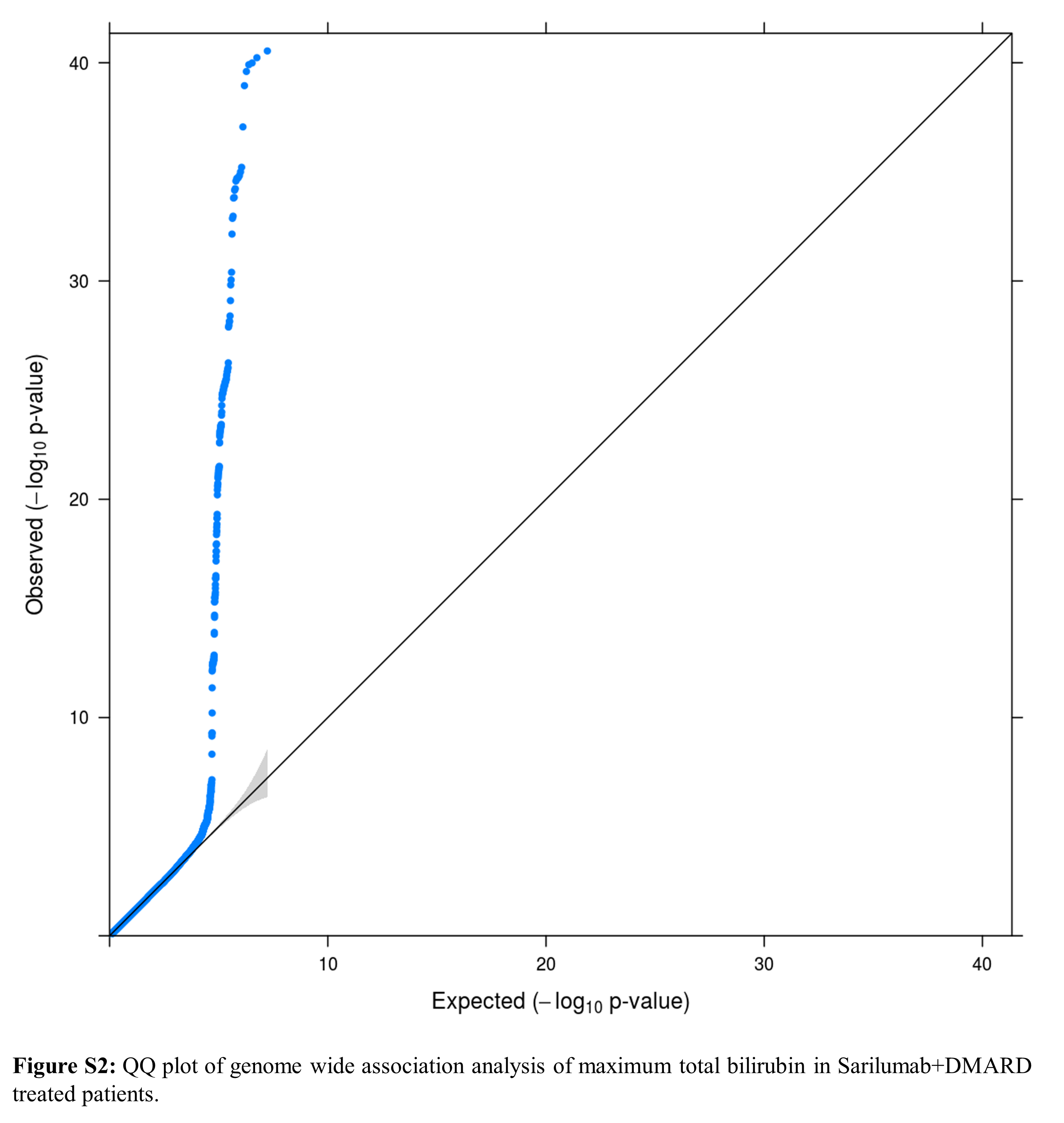

Supplement: Supplementary file 3 — Supplementary figure 2 [file 41397_2022_269_MOESM3_ESM.tif]

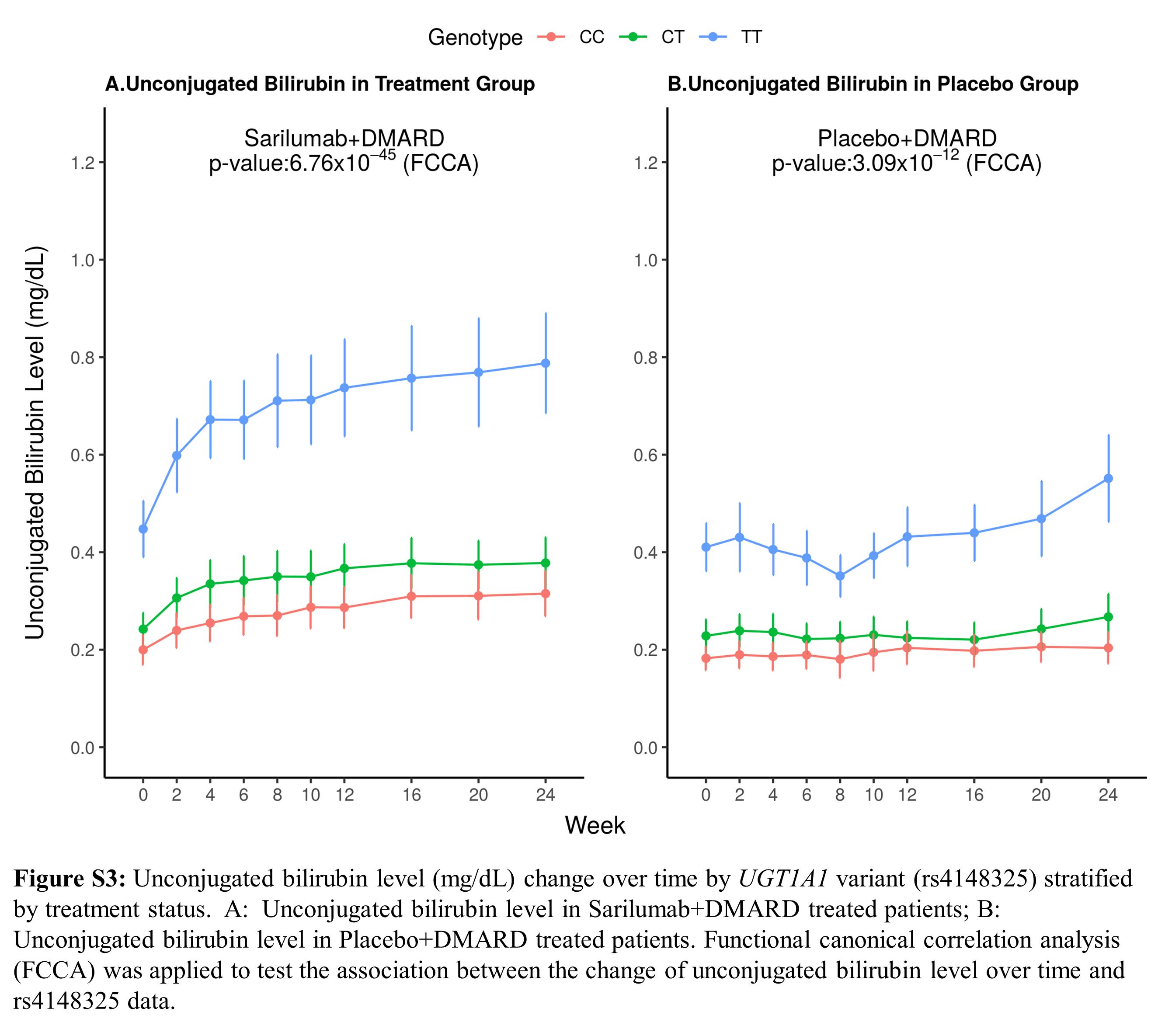

Supplement: Supplementary file 4 — Supplementary figure 3 [file 41397_2022_269_MOESM4_ESM.tif]

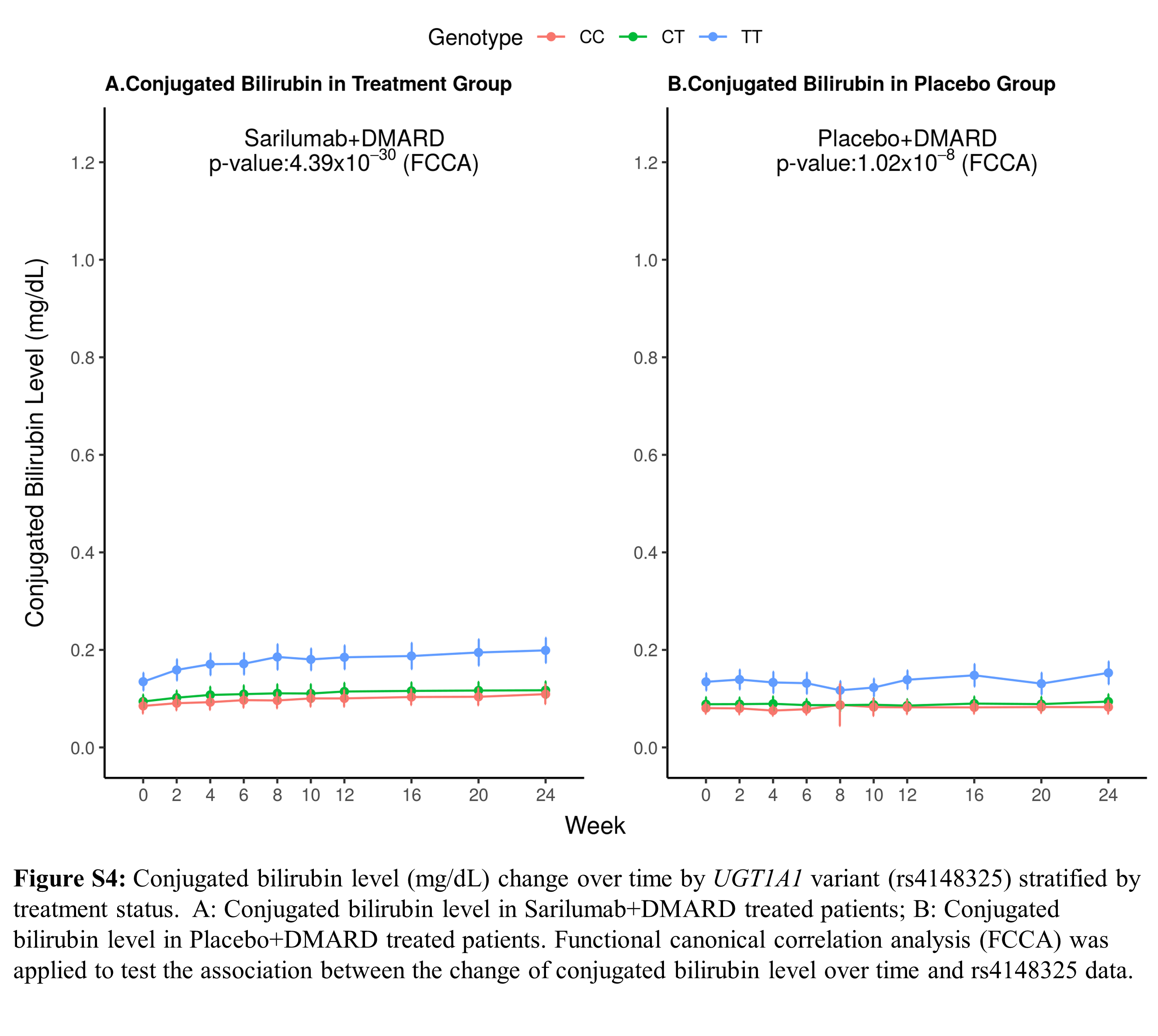

Supplement: Supplementary file 5 — Supplementary figure 4 [file 41397_2022_269_MOESM5_ESM.tif]

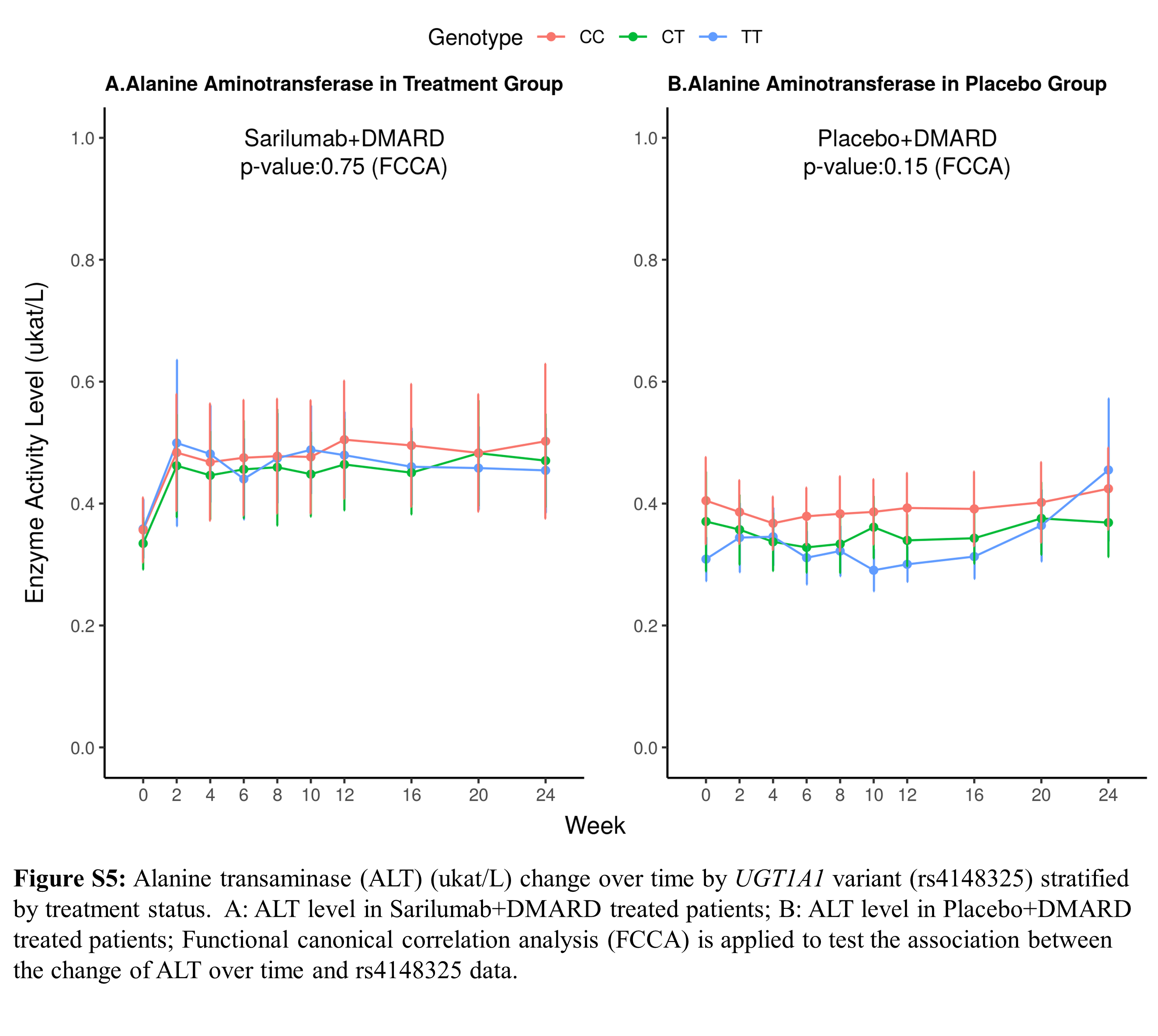

Supplement: Supplementary file 6 — Supplementary figure 5 [file 41397_2022_269_MOESM6_ESM.tif]

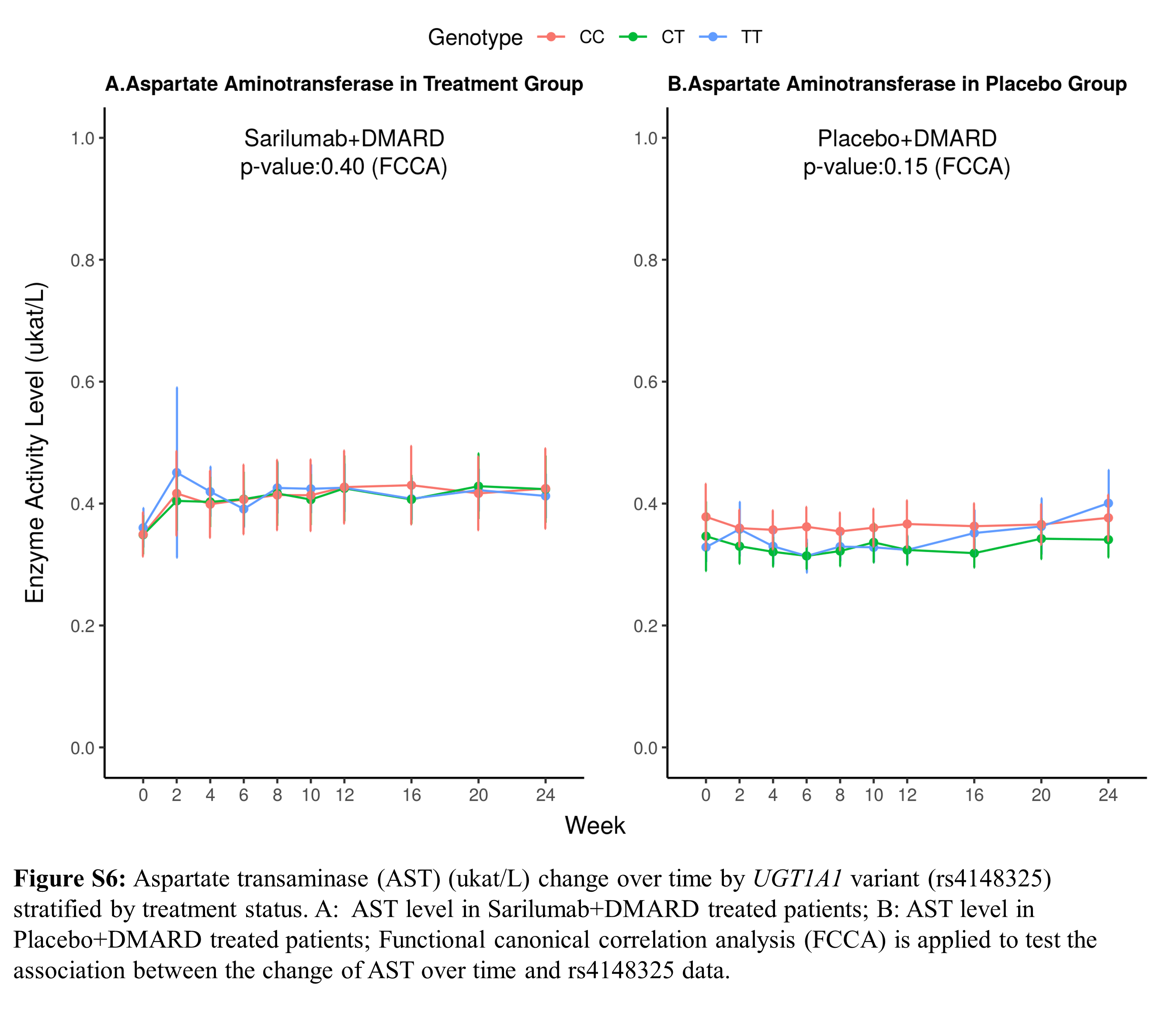

Supplement: Supplementary file 7 — Supplementary figure 6 [file 41397_2022_269_MOESM7_ESM.tif]
